# The impact of language barriers on patients’ perception of their physician’s involvement in shared decision-making

**DOI:** 10.3389/fpubh.2025.1728046

**Published:** 2026-01-13

**Authors:** Sumera Nisar, Saleha Khan, Aseef Rehman, Razan Adel Gobis, Mohammed Shaikhomer, Asim M. Alshanberi, Nahla H. Hariri, Maram H. Alshareef, Safaa M. Alsanosi

**Affiliations:** 1 Department of Ophthalmology, General Medicine Practice Program, Batterjee Medical College, Jeddah, Saudi Arabia; 2 General Medicine Practice Program, Batterjee Medical College, Jeddah, Saudi Arabia; 3 University College of Medicine and Dentistry, University of Lahore, Lahore, Pakistan; 4 Department of Internal Medicine, Faculty of Medicine, King Abdulaziz University, Jeddah, Saudi Arabia; 5 Department of Community Medicine and Pilgrims Health Care, Faculty of Medicine, Umm Al Qura University, Makkah, Saudi Arabia; 6 Department of Pharmacology and Toxicology, Faculty of Medicine, Umm Al Qura University, Makkah, Saudi Arabia

**Keywords:** communication, language barriers, patient perception, Saudi Arabia, shared decision-making

## Abstract

**Background:**

Effective communication is essential in healthcare, particularly when involving patients in treatment decisions. In multilingual settings like Saudi Arabia, language barriers may influence patients’ perceptions of their involvement in shared decision-making. This study explores how language barriers affect patients’ perceptions of their physician’s involvement in treatment decisions in Jeddah.

**Objective:**

To assess how language barriers affect patients’ satisfaction with their physician’s involvement in shared decision-making and to identify key communication-related factors influencing these ratings.

**Methods:**

This cross-sectional study was conducted in Jeddah, Saudi Arabia, from July to October 2024. Adult patients from diverse linguistic backgrounds receiving care at multiple hospitals were included. A total of 410 responses were collected via an online questionnaire, with 399 included in the final analysis. The survey assessed language barriers, patients’ ratings of physician behaviors related to involvement in decision-making, and satisfaction with involvement in care decisions. Descriptive statistics were used, and associations were analyzed using Chi-square (*χ*^2^) tests.

**Results:**

Language barriers were significantly associated with lower ratings of physician involvement in decision-making according to rh patient (*p* < 0.001). Only 17% of patients with communication difficulties rated involvement as “Very Good,” compared to 72% without barriers. Among patients who never felt understood, 74.3% gave poor ratings. Positive ratings were strongly associated with physician listening, politeness, and clear explanations (*p* < 0.001). Saudi nationals were more likely than non-Saudis to rate involvement as “Very Good” (43.5% vs. 23.3%; *p* < 0.001).

**Conclusion:**

Language barriers significantly impact patients’ satisfaction with their physician’s involvement in shared decision-making. Enhancing communication through training and interpretation services can improve patient engagement and overall care quality.

## 1 Introduction

Effective communication between doctors and patients is fundamental to safe and high-quality healthcare. Beyond diagnosis and treatment, clear communication plays a central role in shared decision-making, where patients are involved in choosing among treatment options based on clinical evidence and personal preferences. Language barrier can cause miscommunication, leading to adverse outcomes, including diagnostic errors, non-compliance, and compromised patient satisfaction (1). An estimated one-third of Saudi Arabia’s population consists of foreigners, with the healthcare sector being heavily staffed by non-native Arabic speakers (2). English is the primary language for medical communication and documentation among healthcare professionals; however, most patients prefer Arabic as their preferred language of communication (3).

Physicians in Saudi Arabia stated several challenges due to these language barriers. These include difficulty obtaining accurate patient histories, increased risk of misdiagnosis, limited patient understanding of treatment plans, and non-adherence to medications (4). Many healthcare workers have reported that these challenges contributed to increased adverse clinical outcomes (5, 6).

Previous research has shown that many healthcare professionals recognize these challenges and support measures to overcome them. Several studies highlight that doctors and nurses value the introduction of professional medical interpreters to bridge communication gaps. They also emphasize the importance of structured training programs that teach healthcare workers how to manage language discordance effectively, ensuring that both clinical accuracy and patient comfort are maintained (1, 7). Despite these findings, most available studies in Saudi Arabia have focused on healthcare providers’ viewpoints. There is still limited research examining how patients themselves experience and interpret these language barriers. Very few studies have explored how patients perceive their physicians’ involvement in treatment decisions when effective communication is hindered. Understanding patients’ perspectives is crucial because it reflects the quality of shared decision-making and helps identify areas where communication strategies can be improved.

Hence, the present study aims to explore how language barriers affect patients’ ratings of physician’s involvement in shared decision-making during treatment decisions and their overall satisfaction with healthcare services in Jeddah, Saudi Arabia.

## 2 Materials and methods

### 2.1 Study design, setting and participants

Following the STROBE checklist for cross-sectional studies, this study was conducted to assess the impact of language barriers between healthcare providers and patients. The study took place in Jeddah, Saudi Arabia, from July 2024 to October 2024. Saudi Arabia’s healthcare system includes public, private, and ministry-affiliated hospitals, providing care to both Saudi and non-Saudi residents.

The study included adult patients, both male and female, from various linguistic backgrounds who had received healthcare services in Jeddah across public, private, ministry-affiliated hospitals, and polyclinics. Individuals under the age of 18, those with severe cognitive impairments, and participants with incomplete survey responses were excluded. Hospitals were not individually selected. Instead, the study targeted patients who had received care at any healthcare facility in Jeddah. Data were collected using an online self-administered questionnaire. A questionnaire was developed using Google Drive Forms and disseminated via social media platforms (e.g., WhatsApp), and email. Participants were approached indirectly through these platforms and voluntarily chose to participate. The survey was distributed online rather than at the point of care, so participants completed it at different times after their healthcare encounter. The time since the visit was not standardized and it likely ranged from a few days to several months.

The participants were selected through non-probability convenience sampling. The minimum required sample size was determined to be 350 using Raosoft, based on a 95% confidence level, a margin error of ≤5%, and a likely prevalence of 50%. A total of 410 responses were recorded with a response rate of 99.8%. Any missing data were subsequently excluded from the final analysis.

### 2.2 Survey tool

A questionnaire was designed to assess patients’ views on how language barriers affect their understanding of physician involvement in decision-making. It aimed to gather insights into patients’ perceptions, highlighting how communication gaps may influence their sense of inclusion in care decisions and their overall interaction with healthcare providers. The full questionnaire can be found in Supplementary material S1.

The instrument was structured into three separate sections which consisted of multiple choices, yes/no, and one short answer questions and Likert-type scale questions that were marked as 1 = strongly disagree, 2 = disagree, 3 = neutral, 4 = agree, and 5 = strongly agree. The first section focused on collecting demographic data of the patients such as age, gender, nationality, and mother tongue. The second section aimed to explore language barriers and challenges. This included healthcare provider’s nationality, the use of translators within a healthcare setting, and patient and practitioner’s preferred language of communication. Furthermore, participants were asked about communication difficulties and misunderstandings related to language barriers, and how these issues affected overall patient care.

The third and final section was used to assess patient’s satisfaction, physician-patient interaction during treatment planning, understanding of medical conditions, and healthcare professional’s listening ability and politeness.

### 2.3 Pilot testing

Before commencing the main data collection (1), a pilot study was conducted using a sample of 20 individuals to assess the questionnaire’s reliability and validity. The results from this study showed acceptable content and construct validity, along with adequate internal consistency. Cronbach’s alpha for the overall questionnaire was 0.82, indicating good reliability. Based on certain feedback, minor revisions in phrasing and layout were made to enhance the clarity of the questions.

To maintain proper cultural context and accuracy, the content of the questionnaire was translated by a native speaker proficient in both the Arabic and English languages. Expressions were ensured to be culturally appropriate and naturally phrased for clear understanding. Furthermore, a back-translation was performed into the original language to identify and resolve any mistakes or misinterpretations in the original translation. Lastly, the final version of the data collection instrument was reviewed by two bilingual experts.

### 2.4 Ethical considerations

This study was approved by the ethical review committee of Batterjee Medical College (BMC), Jeddah, Saudi Arabia. Informed consent was obtained from all participants, ensuring voluntary participation, confidentiality, and the right to withdraw at any time. The research team maintained independence and impartiality to avoid conflicts of interest.

### 2.5 Data analysis

The dataset was manually screened to exclude entries with missing demographic information before analysis. Descriptive statistics, including frequencies and percentages, were calculated. Descriptive analyses of the data were represented in tables and graphs in terms of percentages. The Chi-square (*χ*^2^) test was applied to examine the association between nationality (Saudi vs. non-Saudi) and patient perceptions of physician involvement in treatment decisions. Analysis was performed with SPSS “Statistical Package of Social Sciences” (version 27).

## 3 Results

### 3.1 Demographic

A total of 410 participants took part in the study. However, 11 respondents were excluded from the analysis due to incomplete data, resulting in a final sample size of 399 participants. Of these, 160 (40.1%) were male and 239 (59.9%) were female. Participants ranged in age from 13 to 68 years. The sample comprised both Saudi and non-Saudi individuals. Regarding language, 250 (62.7%) spoke Arabic, and 149 (37.3%) spoke languages other than Arabic. When asked whether their healthcare provider communicates in their primary language, 318 (79.7%) responded “Yes,” while 81 (20.3%) responded “No.” Detailed demographics are summarized in Table 1.

**Table 1:** Demographics of participants.

Characteristic	Subgroup	*n* (%)
Gender	Male	160 (40.1%)
	Female	239 (59.9%)
Age Group (in years)	18–19	110 (27.6%)
	20–35	170 (42.6%)
	36–50	75 (18.8%)
	51–68	44 (11.0%)
Nationality	Saudi	180 (45.1%)
	Non-Saudi	219 (54.9%)
Language Spoken	Arabic	250 (62.7%)
	Other than Arabic	149 (37.3%)
Provider communicates in primary language	Yes	318 (79.7%)
	No	81 (20.3%)

Non-Saudi respondents represented multiple nationalities, including South Asian, Southeast Asian, and Middle Eastern regions. Languages other than Arabic included English, Urdu, Hindi, Malayalam, Filipino and Swahili.

### 3.2 Patient perception of physician’s involvement in treatment decisions and influencing factors

A significant association was observed between language barriers and patients’ ratings of their physician’s involvement in treatment decisions (*p* < 0.001). Among participants reporting that language barriers hindered understanding, 13 (10.2%) rated physician involvement as Poor, 49 (38.6%) as Satisfactory, 44 (34.6%) as Good, and 21 (16.5%) as Very Good (*n* = 127). Conversely, those who experienced no impact from language barriers (*n* = 96) reported more favorable ratings, with 72% indicating Good or Very Good involvement. Among participants without any language barrier (*n* = 176), the majority (126; 72%) rated their physician’s involvement as Good or Very Good. Participants who reported feeling consistently understood by their physician (“Always,” *n* = 111) gave markedly higher ratings for physician involvement, with 63 (56.8%) selecting Good or Very Good. In contrast, those who never felt understood (*n* = 136) were significantly more likely to rate physician involvement as Poor or Satisfactory (74.3%). This relationship was statistically significant (*p* < 0.001).

Regarding assessing medical conditions, patients who rated their doctor as “Very Good” in assessment rated the doctor as “Very Good” at involving them in treatment decisions (102; 75.6%). On the other hand, patients who felt their doctor was “Poor” at assessing their condition mostly gave a “Poor” rating for involvement in decision-making (10; 58.8%). Moreover, the perceived politeness of physicians was strongly correlated with involvement ratings. When asked to directly evaluate their physician’s involvement in treatment decisions, 278 participants rated it as Very Good (69.7%), while 121 rated it as Good (30.3%). Respondents who selected Very Good also tended to report higher scores in physician listening, politeness, and mutual understanding (*p* < 0.001), indicating a consistent internal pattern across dimensions of the physician-patient interaction, as shown in Table 2.

**Table 2:** Patient perceptions of doctor involvement based on communication factors and provider behaviors.

Factor	How would you rate your healthcare provider’s involvement of you in decisions about your treatment?					
	Poor	Satisfactory	Good	Very good	Total	*p*-value
To what extent do language barriers impact your understanding of medical care?						
They hinder my understanding	13 (10.2%)	49 (38.6%)	44 (34.6%)	21 (16.5%)	127 (31.8%)	<0.001
They have no impact	0	24 (25.0%)	39 (40.6%)	33 (34.4%)	96 (24.1%)	
They are not applicable (no language barrier)	8 (4.5%)	42 (23.9%)	56 (31.8%)	70 (39.8%)	176 (44.1%)	
How often do you feel that your healthcare provider truly understands you and your concerns?						
Always	9 (8.1%)	39 (35.1%)	40 (36.0%)	23 (20.7%)	111 (27.8%)	<0.001
Often	3 (8.8%)	16 (47.1%)	9 (26.5%)	6 (17.6%)	34 (8.5%)	
Rarely	5 (4.2%)	36 (30.5%)	44 (37.3%)	33 (28.0%)	118 (29.6%)	
Never	4 (2.9%)	24 (17.6%)	46 (33.8%)	62 (45.6%)	136 (34.1%)	
How would you assess your doctor’s politeness in communication?						
Impolite	17 (14.2%)	72 (60.0%)	25 (20.8%)	6 (5.0%)	120 (30.1%)	<0.001
Polite	4 (1.4%)	43 (15.4%)	114 (40.9%)	118 (42.3%)	279 (69.9%)	
Do you feel your doctor listens carefully to what you say?						
No	17 (15.9%)	65 (60.7%)	22 (20.6%)	3 (2.8%)	107 (26.8%)	<0.001
Yes	4 (1.4%)	50 (17.1%)	117 (40.1%)	121 (41.4%)	292 (73.2%)	
How do you perceive your doctor’s role in making decisions about your care?						
Good	17 (14.0%)	78 (64.5%)	21 (17.4%)	5 (4.1%)	121 (30.3%)	<0.001
Very good	4 (1.4%)	37 (13.3%)	118 (42.4%)	119 (42.8%)	278 (69.7%)	

Lastly, overall satisfaction was significantly higher among patients who rated their doctor as “Very Good” at involving them in treatment decisions. Of these, 26 rated their healthcare experience as “Very Satisfactory” and 69 selected “Not applicable” (indicating no language barrier). In contrast, patients who felt their doctor was “Poor” at involving them in decisions were more likely to report dissatisfaction, with 8 rating their experience as “Unsatisfactory” and 1 as “Poor.” This relationship was confirmed by a Pearson Chi-Square value of 38.446 (*df* = 15, *p* < 0.001), indicating a statistically significant association.

### 3.3 Nationality influences perceptions of doctor involvement in decision making

**Figure 1:**
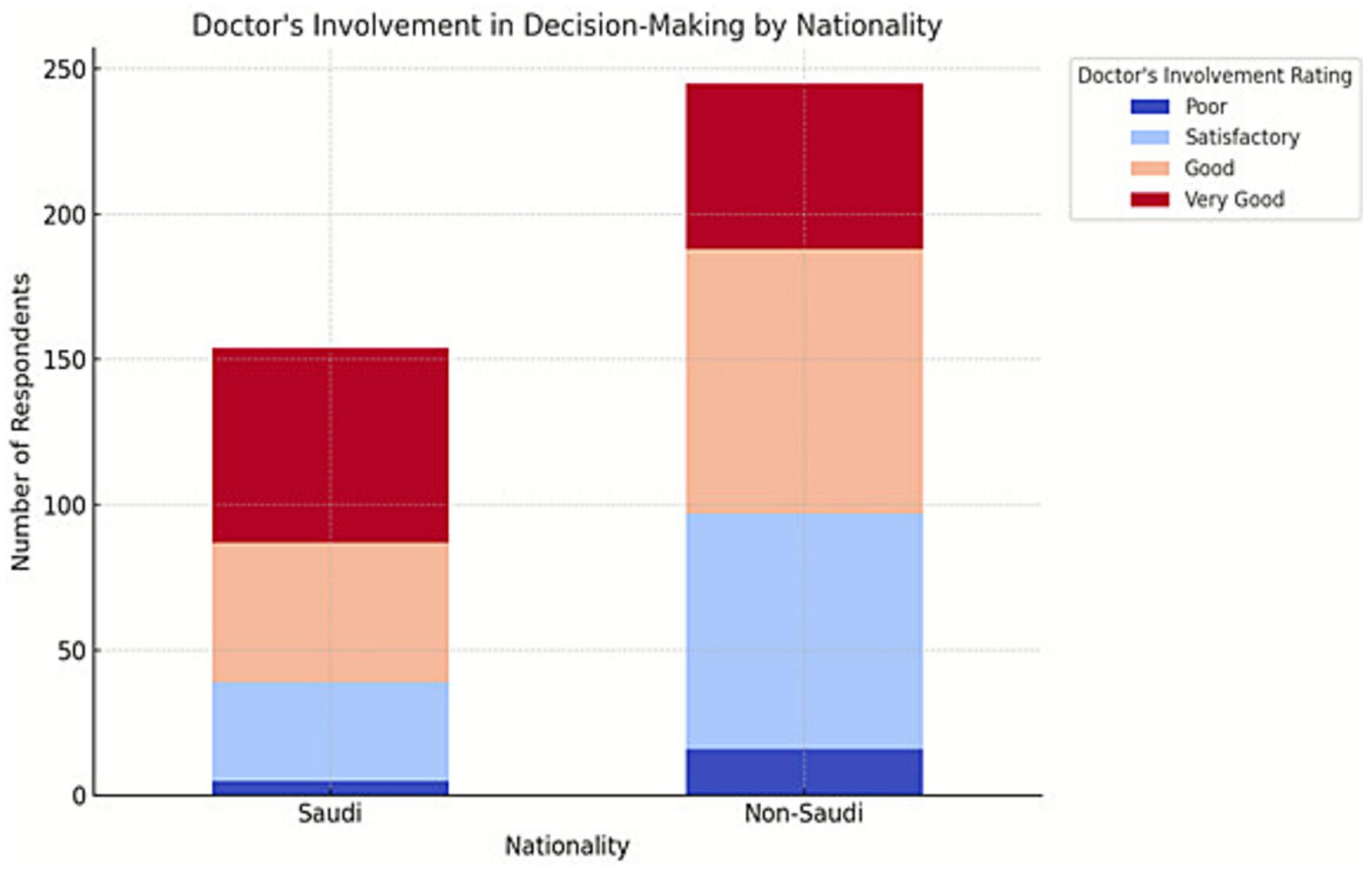
Nationality and patient involvement in decision making.

Saudi patients were more likely to report high satisfaction with doctor involvement in decision making, with 43.5% rating it as “Very Good” compared to 23.3% of non-Saudis. Non-Saudis gave more “Satisfactory” and “Poor” ratings as shown in Figure 1. This difference was statistically significant (*χ*^2^ = 19.330, *p* < 0.001) and may be due to communication barriers, cultural differences, or how doctors engage with patients from different backgrounds.

## 4 Discussion

Saudi Arabia is a multilingual and multicultural society, with a significant proportion of healthcare users and providers coming from diverse linguistic backgrounds. This study investigated how language barriers impact patients’ satisfaction with their physician’s involvement in treatment decision-making, revealing several important associations between communication quality, language discordance, and perceived engagement in care.

Our study found that when patients did not experience language barriers, they generally reported being involved in decisions about their treatment. Patients who reported frequent difficulty understanding their healthcare providers due to language differences indicated lower levels of involvement in decision-making. Among those who did not face language challenges, many rated physician involvement as very good. This further emphasizes the importance of addressing this barrier to improve patient participation. Linguistic barriers can lead to misinterpretations and non-compliance with treatment plans, ultimately affecting patient outcomes (8).

Furthermore, our findings underscore the essential role of both verbal and non-verbal communication skills in fostering patient involvement. Communication elements such as empathy, politeness, and active listening are all linked to enhanced patient satisfaction and perceived involvement in treatment decision-making (9–12). This aligns with prior research from Saudi Arabia and other countries. Empathetic and respectful communication is central to patient-centered care. Other studies have also shown that poor communication, often caused by language barriers, can lead to confusion, misdiagnoses, and strained doctor-patient relationships (13–18).

A particularly strong, statistically significant correlation was observed between physicians’ listening skills and patient involvement. Active listening enhances mutual trust and creates a safe space for patients to express concerns, ask questions, and participate in shared decision-making processes (12, 19). This supports previous evidence that listening is not merely a courtesy but a core clinical skill that influences health outcomes and care satisfaction (18).

Nationality also emerged as a significant variable shaping perception of physician involvement. Saudi patients were more likely to rate physician involvement as “Very Good” compared to non-Saudi patients, a statistically significant difference (*χ*^2^ = 19.330, *p* < 0.001). This disparity may be due to shared linguistic and cultural familiarity between Saudi patients and physicians, or potentially differing expectations of the doctor-patient relationship. Non-Saudi patients may experience greater difficulty navigating healthcare interactions, especially when language services or culturally sensitive communication practices are lacking. A highly significant association was also observed between how patients rated their physicians’ clinical assessment skills and their level of involvement in treatment decisions. Patients who perceived their doctors as “Very Good” at assessing medical conditions were much more likely to report a participatory treatment experience. Conversely, lower ratings of clinical competence correlated with diminished patient involvement.

The data also confirmed a compelling relationship between overall satisfaction and physician involvement. Patients who rated their doctor as “Very Good” in involving them in treatment decisions were significantly more likely to report a “Very Satisfactory” healthcare experience (26 respondents). In contrast, among those who rated physician involvement as “Poor,” 8 reported their experience as “Unsatisfactory” and 1 as “Poor,” indicating a strong statistical association (*p* < 0.001). A systematic review also found that when doctors and patients spoke the same language, overall care was better in 76% of the studies reviewed (20). Similar results were reported in other international studies where various barriers affected patients’ experiences, especially for those with limited English proficiency. When doctors did not offer culturally appropriate care or interpretation, patients felt disconnected, which led to weaker relationships and worse outcomes (21, 22). These findings reflect that communication quality is influenced not only by language proficiency but also by relational factors such as empathy, cultural competence, and interpersonal rapport (22–25). Patients who consistently felt understood, respected, and genuinely heard by their physicians were significantly more likely to report satisfaction and engagement. This insight challenges the narrow framing of language barriers as purely linguistic and suggests a broader framework that includes emotional intelligence and cultural alignment.

Although awareness of these challenges has grown, formal interpreter services and structured communication support are still underused in many healthcare settings (23, 26–29). We recommend targeted training for physicians, increased use of translated materials, and improved communication strategies. Our findings also reinforce the importance of integrating language and communication skills into medical education and expanding access to professional interpretation services within Saudi healthcare. Tailoring communication approaches to individual patient needs, especially among non-Arabic speakers and culturally diverse populations, may enhance both understanding and perceived physician involvement in Saudi Arabia.

## 5 Limitations

While this study provides valuable insights, several limitations should be acknowledged. The reliance on patient self-reported data introduces potential recall bias and subjective interpretation of physician behavior. Additionally, the study sample was geographically limited to healthcare facilities in Jeddah, which may restrict the generalizability of findings to other regions within Saudi Arabia. Moreover, the research focused exclusively on patient perspectives and did not incorporate the experiences or views of healthcare providers. Future studies incorporating both patient and physician perspectives could provide a more comprehensive understanding of communication barriers. Furthermore, because the survey was completed online, participants responded at varying intervals after their healthcare encounters. This lack of standardization in timing may have introduced additional recall bias. Future studies should aim to administer satisfaction surveys consistently and as close as possible to the care encounter to minimize this bias. Finally, convenience sampling was used, which means the sample may not fully represent the wider population, and there is a risk of selection bias. However, this method allowed us to reach a broad range of patients within the practical limits of the study. Future research could include more in-depth interviews and explore factors like culture, family influence, and socioeconomic status that might shape patient involvement in care.

## 6 Conclusion

This study provides robust evidence that language barriers, communication quality, and cultural alignment significantly influence patients’ perceptions of their physician involvement in treatment decision-making. By investing in interpreter services, communication training, and culturally competent care practices, healthcare systems in Saudi Arabia can improve both the effectiveness of treatment and the experience of care for all patients, regardless of linguistic or national background.

## Data Availability

The raw data supporting the conclusions of this article will be made available by the authors, without undue reservation.
