# Supplementary material for: The impact of language barriers on patients’ perception of their physician’s involvement in shared decision-making
Source: Front Public Health. 2026 Jan 13;13:1728046. doi: 10.3389/fpubh.2025.1728046 (PMC12834731; doi:10.3389/fpubh.2025.1728046)
Supplement: Supplementary file 1 [file Table_1.docx]

Supplementary Material- Questionnaire

**Section 1- Consent**

**Section 2- Demographics**

1. What is your age?
2. What is your gender?
3. What is your nationality? *(Please specify)*
4. At which hospital do you usually receive your medical treatment?
5. What language(s) do you speak at home? (English, Arabic, Both English and Arabic, Other)
6. How frequently do you visit your primary healthcare provider?

**Section 3- Language Barrier**

1. Which language do you usually use when communicating with your healthcare provider?
2. Does your healthcare provider communicate in your primary language? (Yes/No)
3. Is your healthcare provider Arab or non-Arab?
4. Do you experience language barriers when communicating with your healthcare provider? (Yes/No)
5. If language barriers are present, how often do you have difficulty understanding your healthcare provider? (Never, Rarely, Sometimes, Often, Always)
6. How would you rate your overall satisfaction with healthcare when language barriers are present? (Poor, Satisfactory, Good, Not applicable [no barriers])\
7. To what extent do language barriers impact your understanding of medical care?
   - They hinder my understanding
   - They have no impact
   - Not applicable (no language barrier)
8. Does your doctor have access to a translator? (Yes/No)
9. Have you ever used a translator during your medical visits? (Yes/No)
10. If yes, how effective was the translator in facilitating communication during your visits?
    - Very effective
    - Effective
    - Neutral
    - Ineffective
    - Very ineffective
11. How important is effective communication to your overall satisfaction with healthcare?
    - Very important
    - Important
    - Neutral
    - Not very important
    - Not important at all

**Section 4- Patient satisfaction**

1. How often do you feel that your healthcare provider truly understands you and your concerns?
   (Always, Often, Sometimes, Rarely, Never)
2. How would you assess your doctor’s politeness in communication?
   (Poor, Satisfactory, Good, Very Good)
3. Do you feel your doctor listens carefully to what you say?
   (Poor, Satisfactory, Good, Very Good)
4. How well do you think your healthcare provider assesses your medical condition?
   (Poor, Satisfactory, Good, Very Good)
5. How do you perceive your doctor’s role in making decisions about your care?
   (Good, Very Good)
6. Overall, how satisfied are you with the healthcare services you receive in Jeddah?
   (Poor, Satisfactory, Good, Very Good)
